# Drug-Induced Hyperthermia Review

**DOI:** 10.7759/cureus.27278

**Published:** 2022-07-26

**Authors:** Michael Horseman, Ladan Panahi, George Udeani, Andrew S Tenpas, Rene Verduzco Jr., Pooja H Patel, Daniela Z Bazan, Andrea Mora, Nephy Samuel, Anne-Cecile Mingle, Lisa R Leon, Joseph Varon, Salim Surani

**Affiliations:** 1 Pharmacy Practice, Texas A&M University, Kingsville, USA; 2 Thermal and Mountain Medicine Division, United States Army Research Institute of Environmental Medicine, Natick, USA; 3 Critical Care, United General Hospital, Houston, USA; 4 Medicine, Texas A&M University, College Station, USA

**Keywords:** serotonin syndrome (ss), thermoprotection, heat stroke, neuroleptic malignant syndrome (nms), malignant hyperthermia (mh), drug-induced hyperthermia, hyperthermia

## Abstract

Humans maintain core body temperature via a complicated system of physiologic mechanisms that counteract heat/cold fluctuations from metabolism, exertion, and the environment. Overextension of these mechanisms or disruption of body temperature homeostasis leads to bodily dysfunction, culminating in a syndrome analogous to exertional heat stroke (EHS). The inability of this thermoregulatory process to maintain the body temperature is caused by either thermal stress or certain drugs. EHS is a syndrome characterized by hyperthermia and the activation of systemic inflammation. Several drug-induced hyperthermic syndromes may resemble EHS and share common mechanisms. The purpose of this article is to review the current literature and compare exertional heat stroke (EHS) to three of the most widely studied drug-induced hyperthermic syndromes: malignant hyperthermia (MH), neuroleptic malignant syndrome (NMS), and serotonin syndrome (SS). Drugs and drug classes that have been implicated in these conditions include amphetamines, diuretics, cocaine, antipsychotics, metoclopramide, selective serotonin reuptake inhibitors (SSRIs), tricyclic antidepressants (TCAs), and many more. Observations suggest that severe or fulminant cases of drug-induced hyperthermia may evolve into an inflammatory syndrome best described as heat stroke. Their underlying mechanisms, symptoms, and treatment approaches will be reviewed to assist in accurate diagnosis, which will impact the management of potentially life-threatening complications.

## Introduction and background

Humans maintain core body temperature via a complicated system of physiologic mechanisms that counteract heat/cold fluctuations from metabolism, exertion, and the environment [1]. Overextension of these mechanisms or disruption of body temperature homeostasis leads to bodily dysfunction, culminating in a syndrome analogous to exertional heat stroke (EHS) [2]. The inability of this thermoregulatory process to maintain the body temperature is caused by either thermal stress or certain drugs [3]. Exertional heat stroke (EHS) is a syndrome characterized by hyperthermia and the activation of systemic inflammation. Several drug-induced hyperthermic syndromes may resemble EHS and share common mechanisms.

The purpose of this article is to review the current literature and compare EHS to three of the most widely studied drug-induced hyperthermic syndromes: malignant hyperthermia (MH), neuroleptic malignant syndrome (NMS), and serotonin syndrome (SS). Their underlying mechanisms, symptoms, and treatment approaches will be outlined in the sections that follow.

Thermoprotection

Similar to mammals with dense hair follicle distribution (i.e., fur) to protect against the sun and insulation (i.e., blubber) to prevent heat loss, the human body has its own specialized integumentary mechanisms to protect against elevated temperatures and overheating [4,5]. Although the human integumentary system has relatively few fat deposits and hair follicles, it is designed to allow heat to escape via thermal radiation [6]. Since human skin is highly vascularized, cutaneous vasodilation allows for unhindered conductive heat loss from the blood [7].

Human skin contains approximately two million eccrine sweat glands [8]. Sweat evaporation transfers heat from the skin surface and blood at differing rates depending on the interplay of various factors, including ambient conditions (i.e., heat and humidity), clothing, exertion, body fluid and osmolality status, body mass composition, and skin surface area [9-11]. Actions such as using a fan or seeking drafts can further cool the skin via convective heat loss and enhanced evaporation [12,13].

Sensory neurons densely innervate the skin, mucosa, and viscera, playing a role in thermoregulation. Specific body temperature ranges are detected by specialized ion channels that respond by activating neurons [14-16]. Some of the more recognized channels are the transient receptor potential (TRP) cation channels [7]. Nine channel types play a role in thermoregulation by helping the body discriminate between cold and hot stimuli [10].

The brain acts as a filter and interprets sensory inputs to create the psychophysical perception of temperature, from cold to cool, to ambient, to warm, to painfully hot [17,18]. In the mammalian brain, bilateral nuclei in the preoptic area of the anterior hypothalamus (POAH) act as a thermostat, monitoring and filtering thermoreceptor input [19]. The POAH then adjusts the core temperature by activating autonomic efferent neuronal pathways, which helps maintain body core temperature to about 1°C [19].

Hyperthermia, fever, and hyperpyrexia

Heat-related disorders are classified and defined in several ways. Some have assigned four different variations to hyperthermia, including fever-induced hyperthermia, exercise-induced hyperthermia, hyperthermia secondary to inadequate means of heat dissipation, and hyperthermia due to impairments of pathological or pharmacological thermoregulatory mechanisms [10].

As described by both the American College of Critical Care Medicine and the Infectious Diseases Society of America, fever is a core body temperature of 38.3°C (100.9°F) or higher, irrespective of the cause [10]. Elevated temperatures may also result from the actions of thermoregulatory pyrogens on the hypothalamus [20]. When the core body temperature rises above 38.3°C (100.9°F) but remains less than 41.5°C (106.7°F), it still falls under the classification of fever [1]. However, once it exceeds 41.5°C (106.7°F), it is known as hyperpyrexia. This condition is most often observed in patients with central nervous system (CNS) injuries (i.e., hemorrhage and cerebral edema) or occasionally in those with severe infections [1].

## Review

Heat stress and heat stroke

**Figure 1:**
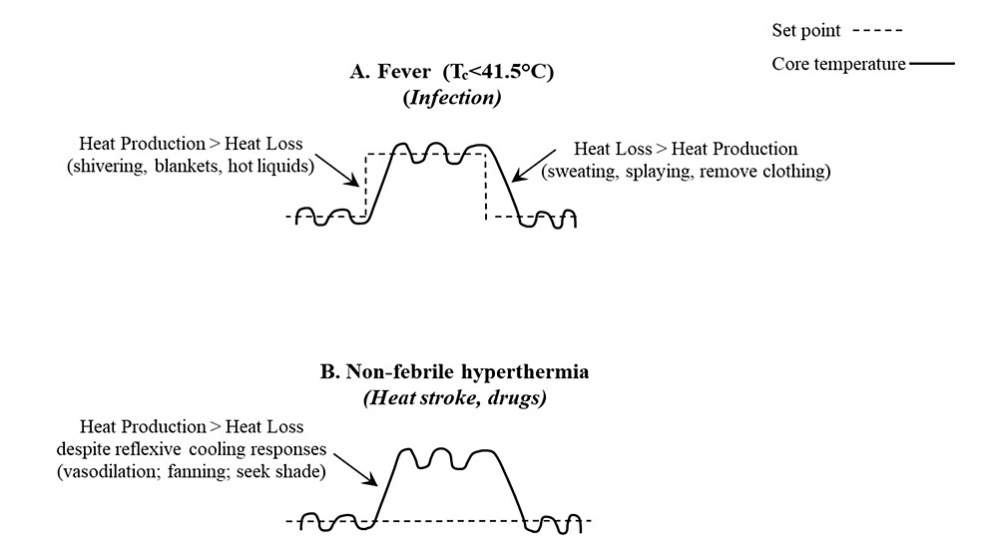
Concept of thermoregulatory changes induced by different environmental stimuli A: Fever is defined as a regulated rise in temperature that is defended by fully functional thermoregulatory mechanisms. Core temperature (Tc) associated with fever is typically <41.5°C. Febrile episodes with Tc>41.5°C are known as hyperpyrexia. B: Non-febrile hyperthermia is characterized by a normothermic setting of the thermoregulatory center in conjunction with an uncontrolled increase in Tc that exceeds the body’s ability to lose heat despite reflexive cooling responses. This occurs with heat stroke and drug-induced hyperthermia. Image credits: Lisa Leon

Heat stress is defined as the net heat load on the body, including metabolic heat production and external environmental factors. More specifically, it represents the sum of generated body heat (metabolic heat), plus temperature gained from the environment (environmental heat), minus body heat lost to the environment (Figure 1). At the cellular level, “heat-shock” proteins (HSPs) act to protect vulnerable proteins and prevent denaturation from repeated heat stress [21]. Positive heat stress may lead to heat strain, defined as the body’s response to a hot environment, especially during physical activity. Patients often perceive heat stress as discomfort [22]. Continuous environmental heat stress, or as a result of exercise, pathological conditions, or drugs, will overcome innate cooling mechanisms, leading up to conditions similar to sepsis and systemic inflammatory response syndrome (SIRS) [23].

Heat stroke is perhaps the most widely studied hyperthermic disorder. It is further classified into two types: classic and exertional (EHS) [12,13]. Pathophysiologic studies have shown it to be a form of SIRS, sharing common mechanistic pathways and clinical features with sepsis [12,13,23]. Similar immune or inflammatory responses may also occur in neuroleptic malignant syndrome (NMS) and malignant hyperthermia (MH), to be discussed later. Severe cases of NMS and MH share clinical symptoms with both heat stroke and sepsis. It seems likely that the body’s response to elevated body core temperature is similar in all hyperthermic disorders. Severe NMS and MH have previously been proposed as neuro-immunological or inflammatory disorders [24-27].

Proposed Mechanisms

Heat stress impacts many other body systems, of which cardiovascular is the most consequential. Heat stress can have several effects on the cardiovascular system. For example, passive heat stress decreases central blood volume due to the redistribution of blood from the central to the cutaneous vasculature, along with increases in cardiac output, heart rate, and contractility [28]. However, during intense exercise, heat stress decreases stroke volume, cardiac output, and muscle blood flow [29].

Classic heat stroke is caused by exposure to high ambient temperatures and occurs more commonly in older patients [30]. The stereotypical patient is an elderly individual with cardiovascular disease living in a home without air conditioning during a heat wave [30]. Conversely, exertional heat stroke is caused by excessive heat generation during physical activity. Although it could occur at room temperature, most cases develop with elevated environmental temperatures. In contrast to classic heat stroke, exertional heat stroke typically occurs in otherwise healthy athletes, military recruits or soldiers, and individuals who work outdoors, especially Caucasian males [12,13,23,31]. Delirium or psychosis with enhanced physical activity can increase endogenous heat production in the absence of high environmental temperatures [32].

**Figure 2:**
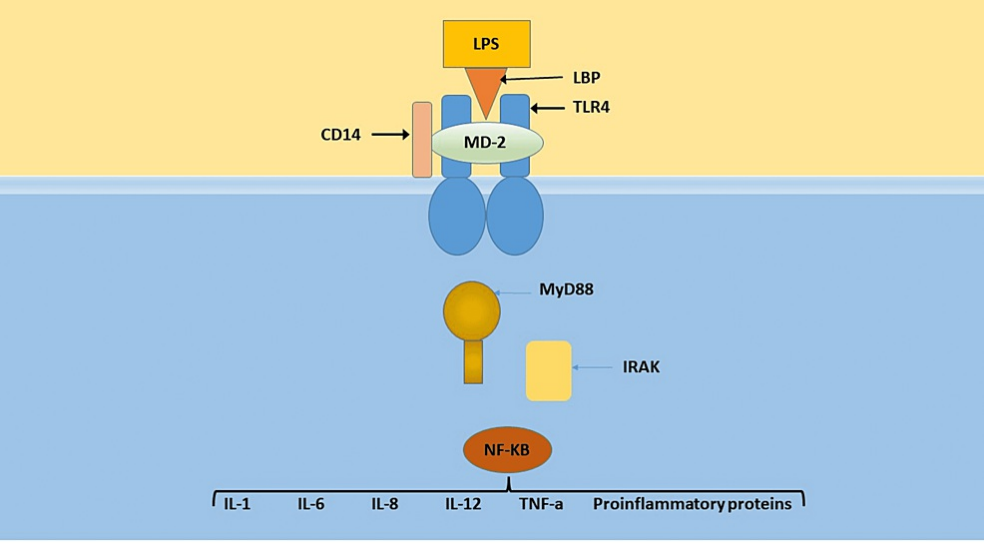
Proposed mechanism of the body’s response to heat stress Image credits: Lisa Leon

In response to heat stress, the sympathetic nervous system reduces blood flow to the splanchnic circulation in order to maintain systemic blood pressure [33]. If prolonged ischemia develops, the tight junctions of the luminal cell layer begin to separate and become “leaky” secondary to nitrosative and oxidative stress [34,35]. This can lead to the translocation of gut bacteria and free endotoxin into the systemic circulation. The lipopolysaccharide (LPS) moiety of endotoxin is a potent pathogen-associated molecular pattern (PAMP) that triggers the inflammatory response in gram-negative sepsis. Circulating endotoxin typically binds to a carrier molecule, including lipopolysaccharide-binding protein (LBP), bactericidal/permeability-increasing protein, soluble CD14, or serum lipoproteins (HDL and VLDL) [36]. LBP is believed to be capable of extracting LPS from bacterial membranes and removing aggregations of free endotoxin in circulation [36]. LBP also enhances the binding or transfer of endotoxin to membrane-bound CD14 receptors on the surface of innate immune cells of monocyte/macrophage lineage. The interaction enables monocytes to respond to low concentrations of LPS (10 pg/mL) [37]. CD14 receptors present endotoxin to the toll-like receptor-4/myeloid differentiation factor-2 (TLR4/MD2) complex. TLR4 transmits a signal facilitated by a series of adapter proteins and kinases including myeloid differentiation factor (MyD88), IL-1 receptor-associated kinase (IRAK), tumor necrosis factor receptor-associated factor-6 (TRAF6), NFқB-inducing kinase (NIK), inhibitor kappa B (IқB), and ultimately nuclear factor-kappa B (NFқB) [38]. NFқB binds to multiple gene promoter regions in the nucleus. The result is a transcription of several hundred genes and the synthesis of clotting elements, complement, other acute-phase proteins, cytokines, chemokines, and nitric oxide. Among the pro-inflammatory cytokines/chemokines synthesized are interleukins (IL-1α, IL-1β, IL-6, IL-8, and IL-12) and tumor necrosis factor-α (TNF-α) [37,39-41]. Figure 2 summarizes the proposed mechanism of the body’s response to heat stress.

Additional inflammatory responses to heat stress include increased transcription of interleukin-6 in muscle cells [42]. It mediates other inflammatory cytokines and stimulates the production of anti-inflammatory acute-phase proteins, which protect against oxidative stress caused by reactive oxygen species (ROS) and proteolytic enzymes [43]. Endothelial cells, epithelial cells, and neutrophils are also involved in acute-phase reactions [13,44-47].

Extreme heat also disrupts membrane fluidity, electrical conductance, and enzyme catalysis [48]. Heat stress increases heat-shock protein (HSP) transcription [13,46,48]. Intracellular proteins are cytoprotective, allowing cells and susceptible tissues to better tolerate thermal stress and thus improve their survival. The extent of the response is directly related to the level of HSP transcription, especially HSP72 (inducible HSP70, also known as HSPA1A or HSP70i), which accumulates in the brain and other tissues [49].

At least four cytoprotective mechanisms for intracellular HSPs have been proposed [50]. The first (and most widely accepted) theory views them as molecular chaperones, binding to folded proteins and preventing detrimental conformational changes due to unfolding or denaturing. A second mechanism proposes that they regulate the central baroreceptor reflex during times of heat stress. A third mechanism proposes an anti-apoptotic mechanism via the inhibition of caspase-dependent apoptosis. The final mechanism proposes that the overexpression of intracellular HSPs, especially the HSP70 family, has an anti-inflammatory effect by inhibiting the production of pro-inflammatory cytokines [13,49,51].

Heat-shock proteins, however, can be a double-edged sword. Extracellular or circulating HSPs, especially eHSP72, are so-called danger-associated molecular patterns (DAMPs). Hepatosplanchnic, vascular and brain tissue, and peripheral blood mononuclear cells serve as the primary sources of eHSP72 [52]. Circulating HSP72 interacts with TLR4 (and TLR2), leading to the transcription and synthesis of the same inflammatory mediators triggered by LPS [53,54]. Levels of circulating HSP72 have been proposed as potential prognostic indicators for heat stroke and other inflammatory disorders such as sepsis [49]. The underlying rationale is that low levels of eHSP72 may be cytoprotective, whereas higher levels augment or exacerbate inflammatory responses [55]. Both endotoxin and extracellular HSP72 appear to contribute to the development of SIRS and multiple organ dysfunction syndrome (MODS).

Several drugs or drug classes can cause or contribute to hyperthermic disorders, including amphetamines, diuretics, cocaine, antipsychotics, metoclopramide, selective serotonin reuptake inhibitors (SSRIs), tricyclic antidepressants (TCAs), and alcohol [32,56,57]. Other drugs such as dopamine agonists and baclofen have been shown to cause hyperthermia upon sudden withdrawal [32,56,57]. These can facilitate heat production or retention via enhanced physical activity or inhibition of cooling [58]. Amphetamines or amphetamine analogs can generate heat by causing psychomotor agitation and impairing pain responses [59]. Additional mechanisms unrelated to physical activity are believed to exist but have not been completely explained [59]. Some have proposed that amphetamines (as well as cocaine and monoamine oxidase inhibitors (MAOIs)) activate the hypothalamic-pituitary-thyroid-adrenal axis, leading to increased levels of circulating thyroid and adrenal hormones or, in the case of PCP and toxic levels of salicylates, uncoupling of oxidative phosphorylation [58,60]. It is also conceivable that these agents may increase hypothalamic concentrations of interleukin-1β, which causes fever [59].

Amphetamines and other sympathomimetic agonists with peripheral vascular α2 effects (e.g., cocaine and ephedra) inhibit conductive cooling by vasoconstriction [61]. Dopamine antagonists may increase the hypothalamic set point or diminish the response of the POAH to heat stress [26]. Drugs with anticholinergic effects inhibit sweating and therefore evaporative cooling. Other drugs such as diuretics or those with diuretic activity (e.g., alcohol) can lead to volume depletion and reduced blood flow to the skin. The result is a decrease in conductive cooling. Drug effects are enhanced in the presence of certain chronic diseases such as ischemic heart disease and diabetes mellitus, obesity, and preexisting infection or inflammation [57,62]. These disorders can also be considered independent risk factors for hyperthermia without the contribution of drugs [57,62]. Cases of heat stroke in which drugs are contributors, but not the direct cause, have been referred to as drug-associated heat stroke [63].

Symptoms

The clinical symptoms of heat stroke can be life-threatening and include acute renal failure, hypotension, or shock, disseminated intravascular coagulation (DIC), encephalopathy, myocardial injury, hepatocellular injury, acute respiratory distress syndrome (ARDS), intestinal ischemia or infarction, and pancreatic injury [12,13,23,57]. The inhibition of dopamine receptors in the POAH may alter the body’s set point [32]. This, in turn, can delay the activation of compensatory responses to heat stress. The rigid, “tetanus-like” skeletal muscle contraction mediated by dopamine blockade in the nigrostriatal tracts leads to heat production [32,62]. Ultimately, the combination of an elevated set point and contraction-generated heat results in hyperthermia. Abnormal calcium metabolism may also contribute to muscular rigidity [64,65].

Heat stroke is characterized by mental status changes with severe hyperthermia (rectal temperature > 40°C or 104°F) [12]. Changes may be subtle but can include impaired judgment, inappropriate behavior, or memory loss. More severe cases may include delirium, obtundation, combativeness, seizures, and coma. Other symptoms include normal blood pressure, hypotension, or shock, disseminated intravascular coagulation (DIC), acute kidney injury, leukocytosis, electrolyte abnormalities, lactic acidosis, dysglycemia, tachypnea, and ARDS. Multiple organ systems can also be affected [12,13,57].

Treatment Approaches

The initial treatment for EHS is to rapidly reduce the patient’s core temperature, which is often accomplished via immersion in either cold or ice water [46,66,67]. Water temperature recommendations vary but typically range between 2°C and 20°C [67,68]. The goal is to achieve a core temperature of ≤39°C (102.2°F) within 30-60 minutes [46,67,69].

Pharmacologic treatment remains controversial. Nonsteroidal anti-inflammatory drugs (NSAIDs), aspirin, and acetaminophen are ineffective and could exacerbate end-organ damage [46,67,69]. Dantrolene is not routinely effective, although small studies suggest benefits in combination with cooling [46,70]. Corticosteroids may reduce pro-inflammatory cytokines. In animal models, the prophylactic use of glucocorticoids prevented heatstroke-induced elevations in LPS. A recent meta-analysis of animal studies found that they reduced morbidity and mortality. However, applying this steroid data to humans remains controversial [70]. Older animal trials evaluated the efficacy of oral prophylactic antibiotics. Kanamycin reduced endotoxemia and cardiac dysfunction without an overall reduction in mortality. Another study found a reduction in mortality with neomycin and tetracycline in combination with laxatives [70].

Neuroleptic malignant syndrome

Proposed Mechanisms

Neuroleptic malignant syndrome (NMS) is a severe adverse effect tied to typical antipsychotics such as haloperidol and fluphenazine [71]. The underlying pathophysiology of NMS is not well understood [24]. The cause is believed to be a blockade of dopamine-D2 or D1 receptors in the corpus striatum and hypothalamus (POAH) [32]. Some authors have challenged this theory, noting case reports of NMS caused by antipsychotics with weak antidopaminergic activity [71]. Regardless, several lines of evidence continue to support the concept of dopamine inhibition, especially the observation that all drugs causing NMS, including those without discernable antipsychotic activity (e.g., metoclopramide), have some degree of dopamine blockade [71]. The abrupt withdrawal of dopaminergic drugs, or rapidly switching from one Parkinson’s medication to another, may also precipitate NMS due to the abrupt withdrawal of D2 receptor stimulation [71]. Additional evidence includes the direct relationship between dopamine receptor affinity and risk of NMS, the effectiveness of dopamine agonists as reversal agents, and clinical similarity between NMS and syndromes characterized by dopaminergic hypoactivity [32,72]. Finally, some authors have proposed similarities to “acute-phase responses,” leading to suggestions that immune or sympathoadrenal dysfunction characterized by loss of hierarchical integration and control may be responsible [24,27,32].

Symptoms

Rigidity is a common presentation of NMS [73]. Patients exhibit catatonic symptoms such as agitation, restlessness, and immobility. Benzodiazepines, especially lorazepam, can be given and continued if symptomatic relief is observed [32,74]. Overall, benzodiazepines may help with rigidity and reduce rhabdomyolysis [73].

Diagnosis

A diagnosis can be made with evidence of exposure to a dopamine antagonist, muscle rigidity, hyperthermia, and at least two of the following: diaphoresis, tremor, altered level of consciousness, elevated or labile blood pressure, tachycardia, elevated creatine phosphokinase, leukocytosis, dysphagia, or incontinence [71,75]. NMS tends to occur within hours to 30 days of starting neuroleptic medications, but symptoms may take up to three days until peak intensity [76].

NMS may present with mild or moderate symptoms including subtle mental status changes [71]. Fever is a defining symptom, but it may not be present in cases associated with atypical antipsychotic agents [74]. The term atypical NMS is used for milder forms or lack of hyperthermia and/or muscle rigidity and inability to meet DSM V criteria [75]. Severe or fulminant NMS is characterized by temperatures ≥ 40°C (104°F) and shares several clinical features with EHS, including mental status changes (ranging from mild drowsiness, agitation, or confusion to a severe delirium or coma), with or without seizures, rhabdomyolysis, lactic acidosis, tachycardia, acute renal failure, leukocytosis, elevated LFTs, and tachypnea [71,75,77]. Blood pressure may be elevated, depressed, or labile [71,75,77]. Most patients are likely to be diaphoretic [71,75,77]. While muscle rigidity is common in NMS, it is typically absent in EHS [77]. Therefore, rigidity may be the only reliable clinical finding to differentiate NMS from EHS in hyperthermic patients taking antipsychotic drugs [32,62,78,79]. Despite differences between NMS and heat stroke, fulminant NMS has been described as a true form of drug-induced heat stroke [32].

Treatment Approaches

Non-pharmacological management includes immediate discontinuation of the inciting medication [74]. When NMS occurs due to the abrupt withdrawal of dopaminergic agents, reinitiating the medication is recommended [71]. Response to conventional antipyretic drugs is poor and poorly established, although physical measures to control temperatures, such as the application of cooling blankets and ice packs, may be helpful, these methods have not been systematically evaluated [75].

Since NMS can be caused by dopamine antagonists, dopamine agonists such as bromocriptine and amantadine may reduce hyperthermia and rigidity while reducing the duration and mortality associated with NMS [74,80,81]. Two to three weeks of bromocriptine therapy may be needed for symptomatic improvement [73]. It also acts as a serotonin agonist, so one must be cautious in differentiating between serotonin syndrome with NMS and avoiding the use of bromocriptine for serotonin syndrome [82].

Concerns arise when rechallenging patients with antipsychotic medications after an NMS diagnosis since such patients require chronic antipsychotic treatment [75]. Some tips for rechallenging include following slower titration patterns, avoiding the administration of parenteral antipsychotic formulations, avoiding the same compound, and re-trialing with atypical antipsychotics with less NMS potential [74,75]. Oral neuroleptics should be rechallenged at least two weeks after symptom resolution, while depot formulations should be re-trialed at least six weeks later [62,71]. In addition, utilizing lower-potency agents at low doses and avoiding combinations with lithium are recommended to reduce the risk of NMS [62,71]. Patients should be instructed to avoid dehydration and counseled and monitored for any symptoms of recurrent NMS [71].

The muscle relaxant dantrolene, which inhibits calcium ion release through the ryanodine receptor isoform 1 in the sarcoplasmic reticulum, can be beneficial in less severe cases of NMS [71,83]. Due to the risk of hepatoxicity, it is typically discontinued once symptoms begin to resolve [62]. Electroconvulsive therapy has reportedly been in refractory cases [71].

Malignant hyperthermia

Proposed Mechanisms

Malignant hyperthermia (MH) can be caused by halogenated anesthetic gasses and succinylcholine. Some afflicted patients exhibit a mutation in the (RYR1) gene. The ryanodine-receptor protein acts as a channel for calcium release from the sarcoplasmic reticulum in striated muscle [84]. Inhaled anesthetics and succinylcholine can prevent the inhibitory effect of magnesium ions in those with the defective (RYR1) gene, leading to unregulated calcium influx and contractions and, if sustained, severe muscular rigidity [65,84-86].

Symptoms

Like NMS, MH is characterized by muscle rigidity and hyperthermia [71,87,88]. Exposure to depolarizing muscle relaxants such as succinylcholine or inhaled anesthetic agents is what separates MH [71]. Another important distinction is the speed of onset and disease progression. MH typically occurs shortly after the induction of anesthesia, although it can occur during any stage of anesthesia and may occur even afterward [87,88].

Symptoms associated with fulminant MH include tachycardia, unstable blood pressure, tachypnea, rhabdomyolysis, acute renal failure, electrolyte abnormalities, coagulopathy, metabolic and respiratory acidosis, pulmonary edema, and death [87,89,90]. Masseter muscle rigidity (MMR), jaw stiffness (due to succinylcholine), and elevated pCO_2_ are often early findings [87,89].

Diagnosis

Hyperthermia can be severe, with temperature increases of 1°C-2°C every five minutes [91]. Maximum temperatures may exceed 41°C (105.8°F) and are associated with a poor prognosis [92].

Treatment Approaches

Early administration of dantrolene inhibits muscle contraction by inhibiting calcium ion release. Several studies demonstrated good response and lower morbidity when it was utilized [84,87,88,91]. Since MMR may precede MH, discontinuation of the triggering anesthetic is advised [91]. Once discontinued, immediate administration of IV dantrolene at a dose of 2.5 mg/kg is recommended. It can be repeated every 15 minutes up to a maximum dose of 10 mg/kg or until the reaction subsides [84,87,88]. When the patient is stabilized, the maintenance dose is 1 mg/kg every 4-6 hours for 24-48 hours following the last observable MH symptoms [87,93].

The toxicity profile of dantrolene includes drowsiness, muscle weakness, pulmonary edema, phlebitis, hepatotoxicity, and seizures [87]. Correction of underlying issues such as hyperthermia, acidosis, hypoxemia, arrhythmias, and preserving renal function must be addressed as well. [88] Calcium channel blockers should be avoided for arrhythmia correction [84,88]. Dantrolene is unnecessary if patients susceptible to MH attempt to avoid triggering agents [91,93]. Discontinuation of triggering agents and close monitoring of patients are emphasized to prevent MH recurrence.

Serotonin syndrome

Proposed Mechanisms

Serotonin syndrome (SS) is caused by supra-therapeutic serotonin levels in the synapses of the brain, and it can lead to hyperthermia [94,95]. This drug-induced condition is often caused by a combination of two or more of the following: SSRIs, monoamine oxidase inhibitors (MAOIs), TCAs, venlafaxine, trazodone, tramadol, linezolid, 3,4-methylenedioxymethamphetamine (MDMA), and several other drugs [95-102]. These agents often share common mechanisms, including the inhibition of serotonin uptake, decreased serotonin metabolism, increased serotonin synthesis, increased serotonin release, activation of serotonergic receptors, and inhibition of cytochrome P450 enzymes [101].

Central and peripheral overstimulation of postsynaptic 5-HT2a receptors is believed to cause severe symptoms such as hyperthermia [101,103,104]. However, the exact mechanism is likely to be multifactorial, involving a complex interplay of excess motor activity and serotonergic-mediated central catecholamine release [105]. The latter causes stimulation of the anterior hypothalamus, leading to the secretion of thyroid hormone and cortisol and the activation of the sympathetic nervous system [105]. The stimulation of sympathetic receptors in brown fat and skeletal muscle can generate heat by upregulating mitochondrial uncoupling protein, resulting in the uncoupling of oxidative phosphorylation [105]. Autonomic-mediated vasoconstriction counteracts the compensatory conductive cooling [60,105-107]. High ambient temperatures could also contribute, especially with MDMA use [60,108].

Symptoms

Compared to NMS, the onset of SS is hyperacute, often within hours, while NMS typically develops over 1-3 days [71,73]. In SS, CNS hyperexcitability is more prominent [73]. Symptoms range from tremors and diarrhea in mild cases to delirium, neuromuscular rigidity, and hyperthermia in life-threatening cases [94]. Myoclonus, hyperreflexia with clonus, and mydriasis tend to be more prominent in SS. Elevated creatine kinase is a clinical feature, but it is also seen in NMS [73]. Symptoms often appear after the initial use of medications, after overdose, or with dosing changes [94,101,109,110]. Table 1 summarizes the clinical presentation and specific characteristics of MH, NMS, and SS.

Diagnosis

**Figure 3:**
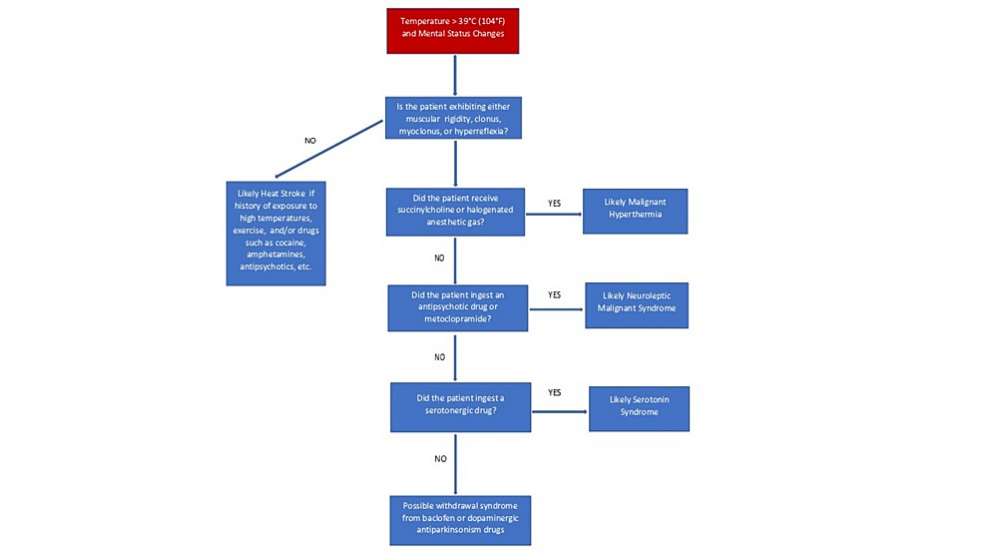
Flowchart to assist in hyperthermia diagnosis Image credits: Michael Horseman

No laboratory test can diagnose SS; therefore, a detailed patient history, physical examination, and knowledge of precipitating drugs can help distinguish it from other syndromes [94]. Clonus (inducible, spontaneous, and ocular) is the most important finding in establishing a diagnosis [111-113]. The degree of muscle rigidity is diagnostic and may be used to separate SS from other hyperthermic syndromes [94]. In addition, mental status changes may appear as agitation or confusion [106]. Mydriasis and diaphoresis are also commonly seen [106]. Figure 3 is a stepwise approach to assist in diagnosing hyperthermia disorders. 

Severe SS can resemble NMS with core temperatures exceeding 41.1°C (106°F) and may include metabolic acidosis, rhabdomyolysis, DIC, elevated transaminases, and acute kidney injury [94,104,106,107,109]. A key differentiator between the two syndromes lies in the fact that bradykinesia with NMS may evolve over several days, while one sees hyperkinesia and a rapid onset with SS [94]. Hyperpyrexia may be quite severe, with temperatures as high as 43.9°C (111°F) [94,104,106,107,109].

Treatment Approaches

Antipyretic agents are largely useless since increases in body temperature are due to muscular activity, not an alteration in the hypothalamic temperature set point [94,114,115]. Diazepam has been studied and is recommended for mild hypertension and tachycardia [101]. It may also reduce temperature [101]. Cyproheptadine, a 5HT-2A receptor antagonist, has been utilized in case reports to provide symptomatic relief in mild to moderate cases [101].

**Table 1:** Characteristics of drug-induced hyperthermia clinical presentation DIC: disseminated intravascular coagulation; LFT: liver function test; MDMA: 3,4-methylenedioxymethamphetamine; SSRI: selective serotonin reuptake inhibitor; POAH: preoptic anterior hypothalamus; SNRI: serotonin and norepinephrine reuptake inhibitor; TCA: tricyclic antidepressants [71,73,84,87,91,115]

Syndrome	Mechanism	Precipitating drugs	Onset	Early clinical features	Severe manifestations
Neuroleptic malignant syndrome	Blockade of dopamine-D2 or D1 receptors in the corpus striatum and hypothalamus (POAH)	Can occur with any antipsychotics but more common with typical antipsychotics, also referred to as first-generation antipsychotics, and can occur in antiemetics such as metoclopramide	Within hours to 30 days of the initiation of antipsychotic medication	Muscle rigidity followed by hyperthermia	Mental status changes (ranging from mild drowsiness, agitation, or confusion to severe delirium or coma) with or without seizures, rhabdomyolysis, diaphoresis, lactic acidosis, tachycardia, acute renal failure, leukocytosis, elevated LFTs, and tachypnea; blood pressure can be high, low, or labile
Malignant hyperthermia	Unregulated calcium influx from the sarcoplasmic reticulum resulting from magnesium ion blockade	Succinylcholine, inhaled halogenated anesthetic gases such as flurane, isoflurane, sevoflurane, desflurane, and enflurane	Shortly after the induction of anesthesia but can occur postoperatively	Masseter muscle rigidity (jaw stiffness) in response to succinylcholine and elevated pCO_2_	Tachycardia, unstable blood pressure, tachypnea, rhabdomyolysis, acute renal failure, electrolyte abnormalities, coagulopathy including DIC, metabolic and respiratory acidosis, pulmonary edema, and death
Serotonin syndrome	Central and peripheral serotoninergic overstimulation of 5-HT2a receptors	SSRIs, linezolid, TCAs, tramadol, SNRIs, trazodone, and MDMA	Within hours of ingesting the offending drugs	Motor and autonomic excitation in conjunction with altered mental status; motor features may include clonus, myoclonus, hyperreflexia, or rigidity, while autonomic effects are usually tachycardia and hypertension; mental status changes may present as agitation or confusion	Core temperatures > 41.1°C (106°F) and metabolic acidosis, rhabdomyolysis, DIC, elevated transaminases, and renal failure

Discussion

Several drugs can produce hyperthermia independently or in combination with high ambient temperatures [12,60,105]. Several mechanisms may be involved affecting either heat generation or compensatory cooling. Drugs causing psychomotor agitation can generate heat from agitation-induced strenuous activity, while agents associated with NMS and MH generate heat from sustained skeletal muscle contraction [32,107,116,117]. Drug-induced hyperthermia may also result from the activation of the pituitary-hypothalamus-thyroid-adrenal axis or the uncoupling of oxidative phosphorylation [60,105,117]. Drugs can affect compensatory mechanisms by stimulating vasoconstriction, thereby reducing blood flow to the skin. This will adversely impact conductive cooling and heat dissipation [60,107]. Cutaneous blood flow can also be reduced by diuretics or other agents capable of decreasing circulating blood volume [118]. Drugs with anticholinergic effects can reduce sweating and therefore adversely impact evaporative cooling [56,61].

Nonetheless, the clinical features of severe or life-threatening cases of drug-induced hyperthermia appear to be similar regardless of the precipitating agent or heat-generating mechanism [119-121]. Signs and symptoms such as elevated temperature, mental status changes, tachycardia, labile or low blood pressure, metabolic acidosis, rhabdomyolysis, DIC, and multisystem organ failure characterize many reports [89,94,107,108,117,120-126]. This suggests that a common pathway may exist in severe cases of drug-induced hyperthermia. It has also been reported, for example, that baclofen withdrawal syndrome can mimic sepsis, a finding that’s not unique to this disorder [119]. Infections including sepsis, meningitis, and encephalitis are often included in the differential diagnosis of other drug-induced hyperthermic syndromes [12,32,89,94,127,128]. Conversely, malignant hyperthermia, neuroleptic malignant syndrome, and serotonin syndrome have been included in the differential diagnosis of sepsis [129]. Gram-negative sepsis is also clinically similar to heat stroke. This is not coincidental since both are considered forms of SIRS. The pathogenesis of gram-negative sepsis and heat stroke involves a PAMP (gram-negative endotoxin or LPS) and a DAMP (circulating HSP72) that interact with toll-like receptor-4 (and TLR-2) [12,13,57]. Either interaction results in the transcription of pro-inflammatory mediators [23,39,40,118,130]. Circulating HSP72 is released not only with heat stress and fever but also with infection including sepsis, acute myocardial infarction, trauma, atherosclerosis, renal failure, sickle cell anemia, hypertension, and other stressors including aging [54,131-133]. Procalcitonin, a marker of the severity of sepsis, bacterial infection, and inflammation (including heat stroke), has been reported to be elevated in case reports of neuroleptic malignant syndrome [134,135]. Procalcitonin is produced and secreted by several tissues in response to lipopolysaccharide (endotoxin) and inflammatory markers such as IL-β, IL-6, TNF-α, and IL-2 [135,136].

All these observations suggest that severe or fulminant cases of drug-induced hyperthermia may evolve into an inflammatory syndrome best described as heat stroke. As mentioned previously, this is not a new concept. The term drug-induced heat stroke has been used before to describe severe NMS and MH [32]. “Exertional heat stroke (EHS)” was used to describe a case series of patients with hyperthermia in which the precipitating agents were determined to be amphetamine analogs (MDMA and MDA) [137].

Unfortunately, there is minimal experimental evidence to support the concept that severe drug-induced hyperthermic syndromes are forms of SIRS. Animal studies of drug-induced hyperthermia have focused on the role of intracellular HSPs and not extracellular HSP72. Nevertheless, there is some indirect evidence suggesting endotoxemia and consequently drug-induced hyperthermia. That evidence is available from in-vitro studies of human Caco-2 cells exposed to heat stress. The Caco-2 cell line is widely used to model the intestinal epithelial barrier [138]. These studies examined the effect of modestly elevated temperature on tight junction (paracellular) permeability. The authors reported a temperature-dependent increase in permeability at 39°C and 41°C. This suggests that elevated temperature alone, independent of the ischemia, may contribute to paracellular leakage of endotoxin [139-141]. Leaked endotoxin has been shown in vivo to magnify tight junction disruption by two mechanisms. One is mediated by the activation of monocytes/macrophages and the subsequent release of pro-inflammatory cytokines such as TNF-α, IL-1β, and INF-γ. The other is mediated by a direct effect on the basolateral membrane of the epithelial wall [142]. Several reports of intestinal ischemia or infarction with methamphetamine and cocaine have been published [143-147]. Two studies, an animal and an in vitro human trial, found that both drugs may disrupt the intestinal barrier and trigger an inflammatory response without inducing hyperthermia [148].

## Conclusions

The human body’s response to heat stress or elevated body core temperature would likely be similar regardless of the heat generating mechanism. It seems likely that the severity of hyperthermia would mirror the level or intensity of eHSP72 release and endotoxemia independent of the etiology. The same compensatory and immunological responses associated with heat stroke probably occur with all drug-induced hyperthermic disorders unless attenuated by the precipitating drug.
